# Advantages of growth and competitive ability of the invasive plant *Solanum rostratum* over two co-occurring natives and the effects of nitrogen levels and forms

**DOI:** 10.3389/fpls.2023.1169317

**Published:** 2023-04-18

**Authors:** Jian-Kun Sun, Ming-Chao Liu, Kai-Qi Tang, En-Xiong Tang, Jian-Min Cong, Xiu-Rong Lu, Zhi-Xiang Liu, Yu-Long Feng

**Affiliations:** ^1^Liaoning Key Laboratory for Biological Invasions and Global Changes, College of Bioscience and Biotechnology, Shenyang Agricultural University, Shenyang, Liaoning, China; ^2^College of Agricultural and Biological Engineering, Taizhou Vocational College of Science & Technology, Taizhou, Zhejiang, China

**Keywords:** advantages, competitive ability, growth, exotic plants, leaf area, nitrogen levels and forms, photosynthesis, root to shoot ratio

## Abstract

**Introduction:**

Atmospheric nitrogen (N) deposition has often been considered as a driver of exotic plant invasions. However, most related studies focused on the effects of soil N levels, and few on those of N forms, and few related studies were conducted in the fields.

**Methods:**

In this study, we grew *Solanum rostratum*, a notorious invader in arid/semi-arid and barren habitats, and two coexisting native plants *Leymus chinensis* and *Agropyron cristatum* in mono- and mixed cultures in the fields in Baicheng, northeast China, and investigated the effects of N levels and forms on the invasiveness of *S. rostratum*.

**Results:**

Compared with the two native plants, *S. rostratum* had higher aboveground and total biomass in both mono- and mixed monocultures under all N treatments, and higher competitive ability under almost all N treatments. N addition enhanced the growth and competitive advantage of the invader under most conditions, and facilitated invasion success of *S. rostratum*. The growth and competitive ability of the invader were higher under low nitrate relative to low ammonium treatment. The advantages of the invader were associated with its higher total leaf area and lower root to shoot ratio compared with the two native plants. The invader also had a higher light-saturated photosynthetic rate than the two native plants in mixed culture (not significant under high nitrate condition), but not in monoculture.

**Discussion:**

Our results indicated that N (especially nitrate) deposition may also promote invasion of exotic plants in arid/semi-arid and barren habitats, and the effects of N forms and interspecific competition need to be taken into consideration when studying the effects of N deposition on invasion of exotic plants.

## Introduction

Exotic plant invasion has become a serious global eco-environmental problem, affecting the species composition, structure and function of invaded ecosystems, and even threatening human health (Zhao et al., 2020; Iqbal et al., 2021; Chen et al., 2022). It is important to explore the key factors determining invasion success of alien plant species for develop effective prevention and management (Lau and Schultheis, 2015; Luo et al., 2022). A high competitive ability is one of the key factors facilitating invasion success of exotic plants in the fields (Qin et al., 2013; Zheng et al., 2015; Liu et al., 2018; Zhang and van Kleunen, 2019; Ba and Facelli, 2022). The higher competitive ability of invasive relative to native plants are associated with their higher resource capture ability and utilization efficiency (Davidson et al., 2011; Feng et al., 2011; Liao et al., 2013; Luo et al., 2019), which are often indicated as higher plant height, specific leaf area, total leaf area, photosynthesis, photosynthetic resource-efficiencies, and many other functional traits (Feng et al., 2007a; Feng et al., 2007b; Leishman et al., 2007; Feng et al., 2011; Luo et al., 2019; Huang et al., 2020; Liu et al., 2022).

The competitive relationships between invasive and native plants are not immutable, and may change with environmental factors or global changes (Liao et al., 2013; Qin et al., 2013). As an essential component of global changes, atmospheric nitrogen (N) deposition gradually enhances soil N availability (Zhou et al., 2019; Cao et al., 2021). Many studies have shown that the increased soil N availability promotes the growth of invasive and native plants, especially for the former, enhancing competitive ability of invasive plants (Liu et al., 2017b; Musso et al., 2021). However, some exotic plant species have higher resource utilization efficiencies, and can invade barren habitats (Liu et al., 2017a). In addition, the effects of N forms on exotic plant invasions have received little attention (Sun et al., 2021; Luo et al., 2022; Zhang et al., 2022).

Ammonium and nitrate are the two main N sources that can be directly absorbed by plants from soils, but their relative contents (proportions) in soils are variable with habitats (Sun et al., 2021). The proportion of nitrate N in atmospheric N deposition is increasing gradually (Liu et al., 2013; Yu et al., 2019), which may affect soil N forms, and then plant growth (Sun et al., 2021). Some plants always prefer to absorb a specific form of soil N in nature, and plants perform better under their preferred N form than under other N forms (Rossiter-Rachor et al., 2009; Vu et al., 2021; Luo et al., 2022; Zhang et al., 2022). For example, total biomass of the invasive plant *Xanthium strumarium*, which prefers nitrate N, was higher under nitrate relative to ammonium N, while those of *Flaveria bidentis* and *X. sibiricum* were lower in nitrate relative to ammonium N (Huangfu et al., 2016; Luo et al., 2022). However, most of the related studies that focused on the effects of N forms on the performance of plants were mainly conducted in monoculture, and little attention was paid to the effects in the fields under competitive conditions. It has been demonstrated that interspecific competition changes plants’ preference for N forms (Sun et al., 2021). In addition, most of the competition experiments between invasive and native plants were conducted in pots under controlled conditions, which is difficult to truly reflect the competitive relationship between plants under natural conditions.

The invasive plant *S. rostratum* and two coexisting native plants *L. chinensis* and *A. cristatum* were compared in this study. *S. rostratum*, an annual herb in Solanaceae, is native to North America and one of the most notorious invasive weeds in northeast China, Inner Mongolia, and Xinjiang. It was first found in Liaoning, northeast China in 1981. It mainly invades barren habitats, especially disturbed grasslands, roadsides and field-sides, seriously affecting the ecological environment and economic development in Liaoning, Jilin and Xinjiang (Feng, 2020). *L. chinensis* and *A. cristatum*, perennial herbs in Poaceae, are the dominant or common species in the steppes of northeast China, Inner Mongolia, and Xinjiang, and *S. rostratum* often infests in the steppes when disturbed.

Due to atmospheric N deposition and other processes, the increased soil N availability and shifted soil N forms may influence the growth and competitive relationships between invasive and native plants, promoting invasion success of exotic plants. Exotic plant invasions have become more and more severe with the increase of atmospheric N deposition (Musso et al., 2021; Luo et al., 2022; Zhang et al., 2022). To investigate the roles of N levels and forms in successful invasions of exotic plants, we compared the invasive plant *Solanum rostratum* Dunal, which prefers nitrate relative to ammonium N (Luo et al., 2022), and the two coexisting native plants *Leymus chinensis* (Trin.) TZvel and *Agropyron cristatum* (L.) Gaertn grown in mono- and mixed cultures under different N levels of nitrate and ammonium N in the field. In this study, we measured the aboveground biomass, total biomass, root to shoot ratio, total leaf area, light-saturated net photosynthetic rate, and competitive ability of each species under each N treatment. We hypothesize that (1) the biomass and competitive ability of *S. rostratum* are higher than *L. chinensis* and *A. cristatum*, which are associated with its stronger resource capture ability; (2) N addition facilitates growth of the invasive and native plants, and its effects are higher for the former, therefore enhancing competitive ability of the invader; (3) growth and competitive ability of *S. rostratum* are higher under nitrate relative to ammonium N.

## Materials and methods

### Study sites and species

This study was conducted at the field abandoned for ≈20 years, which is located in the experimental farm of Baicheng Normal University, Baicheng, Jilin Province (45°10′15.6″ N, 122°49′20.9″ E; asl. 180 m). This region belongs to the temperate continental monsoon climate with long winters and short summers. In this area, the annual mean temperature was 5.2 °C, annual mean precipitation was 399.9 mm (mainly from May to September), and the mean annual sunshine was 2915 h (http://www.jlbc.gov.cn/zjbc_3289/zrdl/dlgk/201612/t20161226_55098.html). The soil (sandy) contained 55.71 g kg^-1^ organic matter, 19.97 g kg^-1^ total potassium, 0.85 g kg^-1^ total phosphorus, 6.13 mg kg^-1^ ammonium N, 35.51 mg kg^-1^ nitrate N, 372.77 mg kg^-1^ available potassium and 70.43 mg kg^-1^ available phosphorus (measured by the Analytical and Testing Center, Shenyang Agricultural University). The soil in the study site was fertile according to the evaluation criteria of soil fertility (Sun et al., 1995). The main vegetation of the study sites included *L. chinensis*, *A. cristatum*, *Setaria viridis*, *S. nigrum*, *Amaranthus apinosus*.

The seeds of *S. rostratum* were collected from multiple plants with more than 20 m apart for one another in Inner Mongolia in autumn of 2020. The seeds of *L. chinensis* and *A. cristatum* were purchased from Shenyang Jinfuyou Seed Co., Ltd (Shenyang, Liaoning Province, China).

### Experimental design

All plants (including rhizomes) were removed from the study site before the experiment (May of 2021). The soil was turned over (25 cm depth) and leveled, and 1.2 m × 1.2 m plots were setup, which were spaced at a distance of 60 cm apart. The invasive and native plants were grown in the plots in mon- and mixed culture, respectively. In monoculture, 81 individuals of each species were cultivated in each plot (9 rows and 9 columns); in mixed culture, 16 individuals of *S. rostratum* were planted with 65 individuals of *L. chinensis* or *A. cristatum* in each plot (9 rows and 9 columns; **Figure S1**). Five N treatments were applied: no N addition (control, CK), low ammonium N (LA; 6 g N m^-2^), low nitrate N (LN; 6 g N m^-2^), high ammonium N (HA; 24 g N m^-2^), and high nitrate N (HN; 24 g N m^-2^). The N fertilizer was added in six sperate applications at an interval of 5 d. In total, 100 plots were built [5 N treatments × 5 planting methods (3 monocultures + 2 mixed cultures) × 4 replicates]. Ammonium N was provided in the form of ammonium chloride (NH_4_Cl), and nitrate N was provided in the form of sodium nitrate (NaNO_3_). Both were added as aqueous solutions, while the control was added with an equal amount of water. The nitrification inhibitor dicyandiamide (9.75 g m^-2^) was added into each plot for inhibiting ammonium N transformed into nitrate N (Zhou et al., 2021). The total amount of N added under low N treatments was set based on the highest atmospheric N deposition in China (Wen et al., 2020).

The seeds of the three plants were stratified in wet sand for 14 d at 4 °C, disinfected with 0.5% potassium permanganate for 30 mins, washed repeatedly with tap water, drained, and then sown at the 81 sites in e plot (15 cm spacing and 0.5 cm depth; **Figure S1**). Ten seeds were sown at each site, and the seedlings were thinned when growing up to about 4 cm, leaving one seedling at each site. The N addition treatments were applied for the first time after one week of thinning. The plots were managed by routine agronomic practice, with manual weeding and automatic irrigation.

### Measurements

In mid-August of 2021, light-saturated photosynthetic rate (*P*_max_) was measured on a recently matured healthy leaf for each species and treatment per plot using a Li-6400 Portable Photosynthesis Meter (Li-Cor, Lincoln, NE, USA). Light intensity in leaf chamber was set to 1500 µmol m^-2^ s^-1^, CO_2_ concentration of reference chamber was set to 380 µmol mol^-1^, leaf temperature was set to 27 °C. The leaves were fully induced under saturated light before measurement.

In early September, one plant per species per treatment per plot was randomly sampled, and aboveground parts were collected and divided into stems and leaves. Total leaf area was measured with a LI-3100C Area Meter (LI-COR, NE, Lincoln, USA), then leaves and stems were dried at 60 °C for 72 h and weighed, respectively. Roots were dug out with a shovel (above 30 cm depth), rinsed with tap water, dried at 60 °C for 72 h, and weighed. Total biomass was calculated as the sum of leaf biomass, stem biomass, and root biomass; aboveground biomass was calculated as the sum of leaf biomass and stem biomass; root to shoot ratio was calculated as the ratio between root biomass and aboveground biomass.

To compare competitive ability of the invasive and native plants, the modified relative competitive intensity (RCI) was calculated (Grace, 1995; Zheng et al., 2015).


RCI =(Pmix−Pmono)/Pmono

Where *P*_mix_ and *P*_mono_ represented aboveground biomass or total biomass for individual plants grown in mixed and monoculture, respectively. RCI = 0 indicates no competition between the two species; RCI > 0 indicates that competing species promotes growth of the target species; RCI< 0 indicates that competing species inhibits growth of the target species.

### Statistical analysis

Effects of species, N forms, N levels, planting methods, and their interactions on total biomass, aboveground biomass, root to shoot ratio, total leaf area, and *P*_max_ were tested using four-way analysis of variance (ANOVA). One-way ANOVA was used to assess the differences in the parameters and RCI between nitrogen treatments for the same species under the same planting method, and the differences between species grown under the same N addition treatment in monoculture. Significances were tested by least significant difference (*p*< 0.05). The differences in above parameters and RCI between the invasive species and each native plant under the same N addition treatment in mixed culture were analyzed using independent samples *t*-test. All statistical analyses were performed using PASW Statistics 18.0 (SPSS Inc., Chicago, IL, USA). Prior to doing statistical analyses, normality and chi-square were checked. Standardized major axis regression (SMA) was used to analyze the relationships between aboveground biomass, total biomass and total leaf area, root to shoot ratio, as well as the differences between the invasive and native plants when grown in monoculture, mixed culture, and both, respectively. Statistical analyses were conducted with R version 3.6.1 (R Development Core Team, Vienna, Austria). Plotting was performed with Sigmaplot version 10.0 (Systat, San Jose, CA, USA).

## Results

### Growth and competitive ability

**Figure 1:**
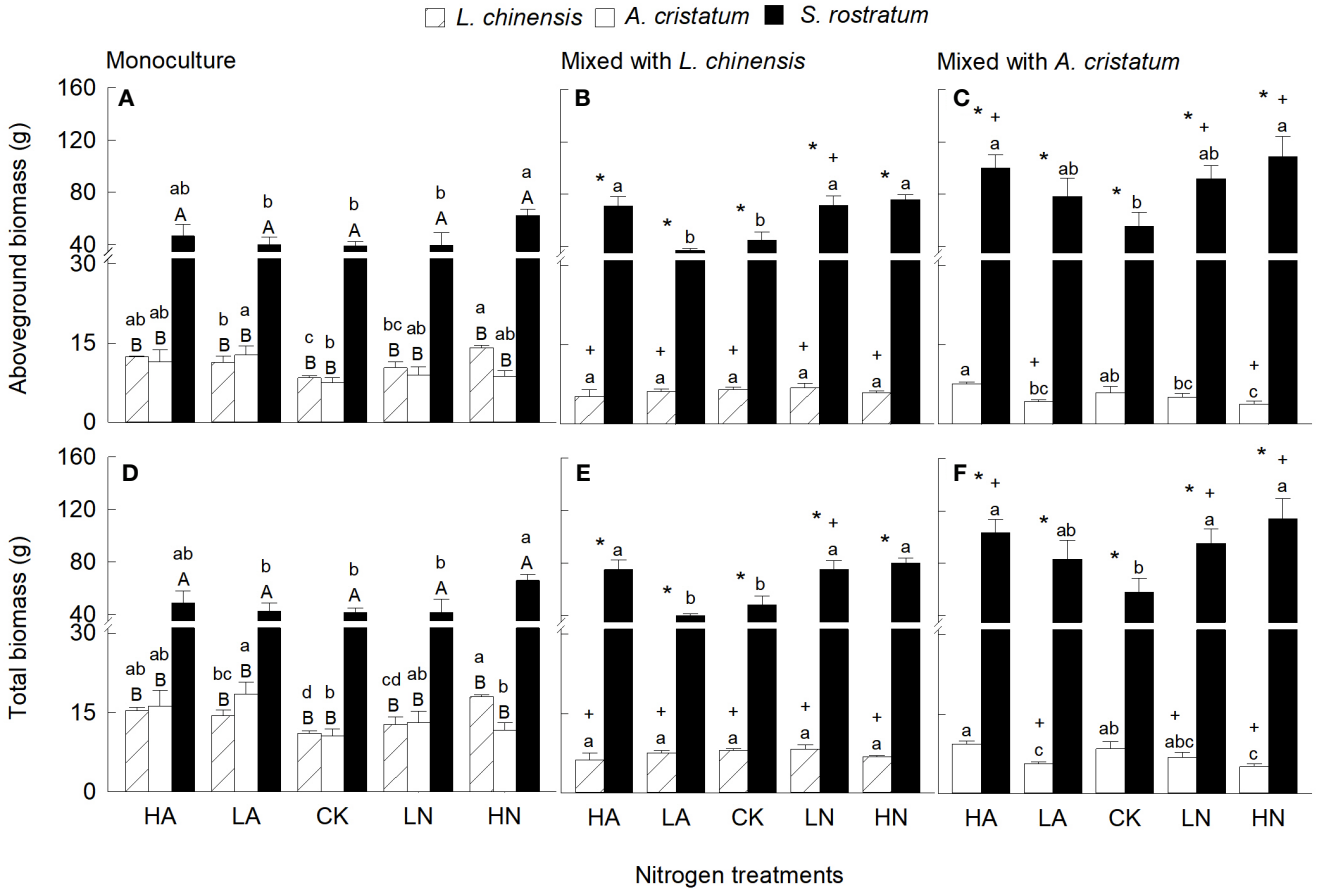
Aboveground biomass **(A–C)** and total biomass **(D–F)** for Leymus chinensis, Agropyron cristatum and Solanum rostratum grown in mono- **(A, D)** and mixed **(B, C, E, F)** cultures under different nitrogen treatments. HA, high ammonium; LA, low ammonium; CK, control; LN, low nitrate; HN, high nitrate. Mean ± SE (n = 4). Different upper- (* in mixed culture; independent samples t-test) and lowercase letters indicate significant differences between species under the same nitrogen treatment and those between nitrogen treatments for the same species under the same planting method, respectively (P < 0.05; one-way ANOVA). + indicates significant difference between the mixed and monocultures under the same nitrogen treatment (P < 0.05, independent samples t-test).

Aboveground biomass and total biomass were significantly higher in the invasive relative to the two native plants in either mixed and monocultures, and the magnitude of the differences increased with increasing N levels in most cases (**Figure 1**). Four-way ANOVA results also showed that aboveground biomass and total biomass were significantly affected by species and their interactions with N levels (**Table S1**). The aboveground biomass and total biomass of *S. rostratum* were higher under low nitrate relative to low ammonium treatment in mixed culture (**Figures 1B, C, E, F**). For *L. chinensis* and *A. cristatum*, however, N forms did not significantly affect aboveground and total biomass under any N level in either mixed or monoculture (except *A. cristatum* under high N level in mixed culture; **Figure 1**). N addition increased aboveground and total biomass of *S. rostratum* in both mixed and monocultures (except competed with *L. chinensis* under low ammonium), but the effects of N addition were not always statistically significant (**Figure 1**). For the two native plants, N addition increased aboveground and total biomass in monoculture, but the effects were not significant in few cases (**Figures 1A, D**). In mixed cultures, however, N addition did not significantly increase the aboveground and total biomass for *L. chinensis*, and even reduced the biomass for *A. cristatum* (**Figures 1B, C, E, F**). Compared with monoculture, mixed culture increased aboveground and total biomass of the invader (except competed with *L. chinensis* under low ammonium), while reduced the biomass of the native plants. These results were consistent with those of our four-way ANOVA (**Table S1**).

**Figure 2:**
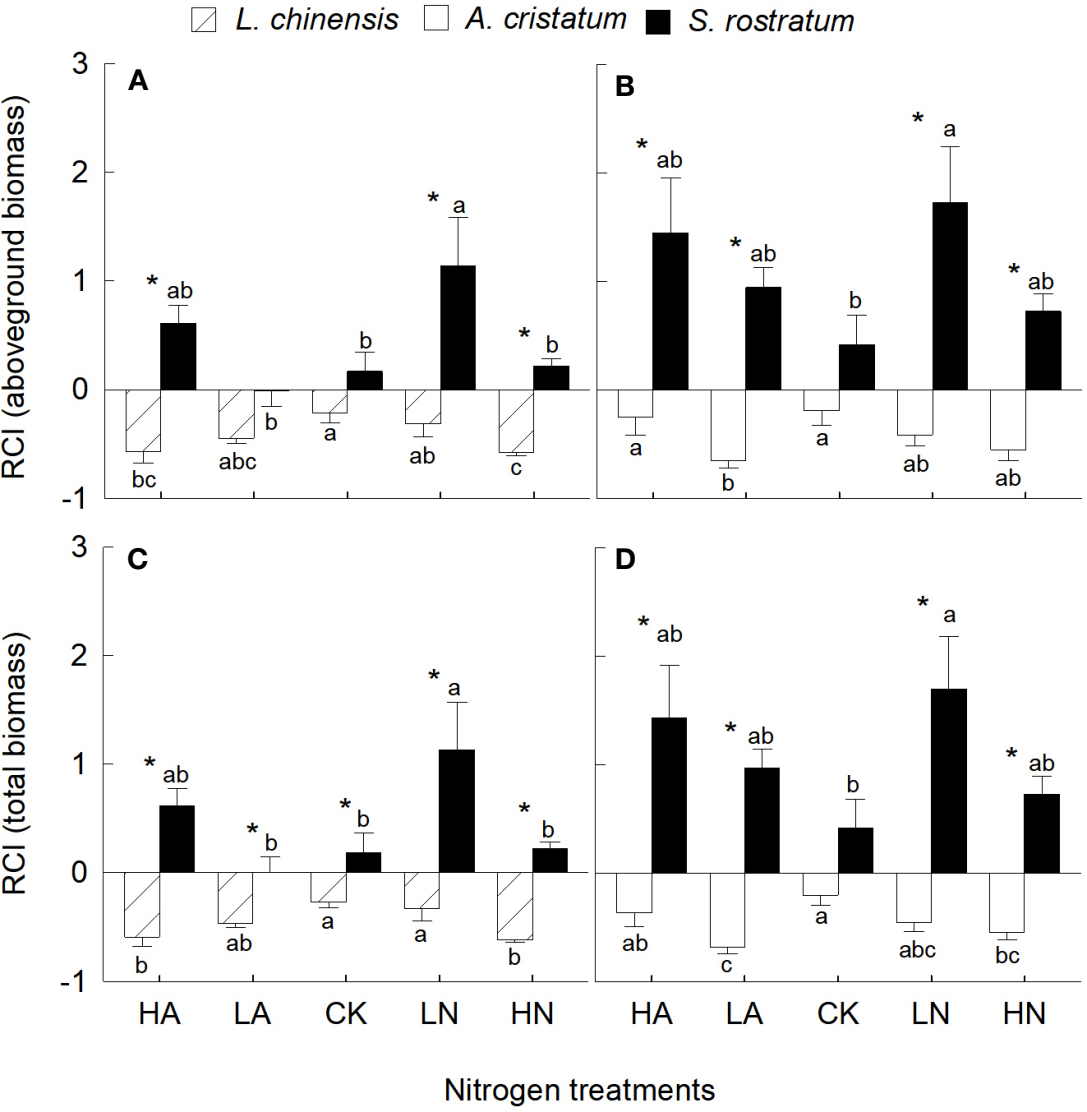
Relative changes of aboveground biomass **(A, B)** and total biomass **(C, D)** in Leymus chinensis, Agropyron cristatum and Solanum rostratum grown under different nitrogen treatments. RCI, relative competition intensity. HA, high ammonium; LA, low ammonium; CK, control; LN, low nitrate; HN, high nitrate. Mean ± SE (n = 4). Different lowercase letters indicate significant differences between nitrogen treatments for the same species (P < 0.05; one-way ANOVA); * indicates significant differences between species under the same N treatment (P < 0.05; independent samples t-test).

In most cases, *S. rostratum* had significantly higher competitive ability than the two native plants based on their aboveground and total biomass (**Figure 2**). In most conditions, N addition enhanced the competitive ability of the invader, while reduced that of the two native plants, thus increased the competitive advantages of the invader over the native plants. For the invader, the competitive ability was significantly higher under low nitrate relative to low ammonium treatment, while the effect of N forms on the competitive ability of the two native plants was not significant.

### Total leaf area, root to shoot ratio and photosynthesis

**Figure 3:**
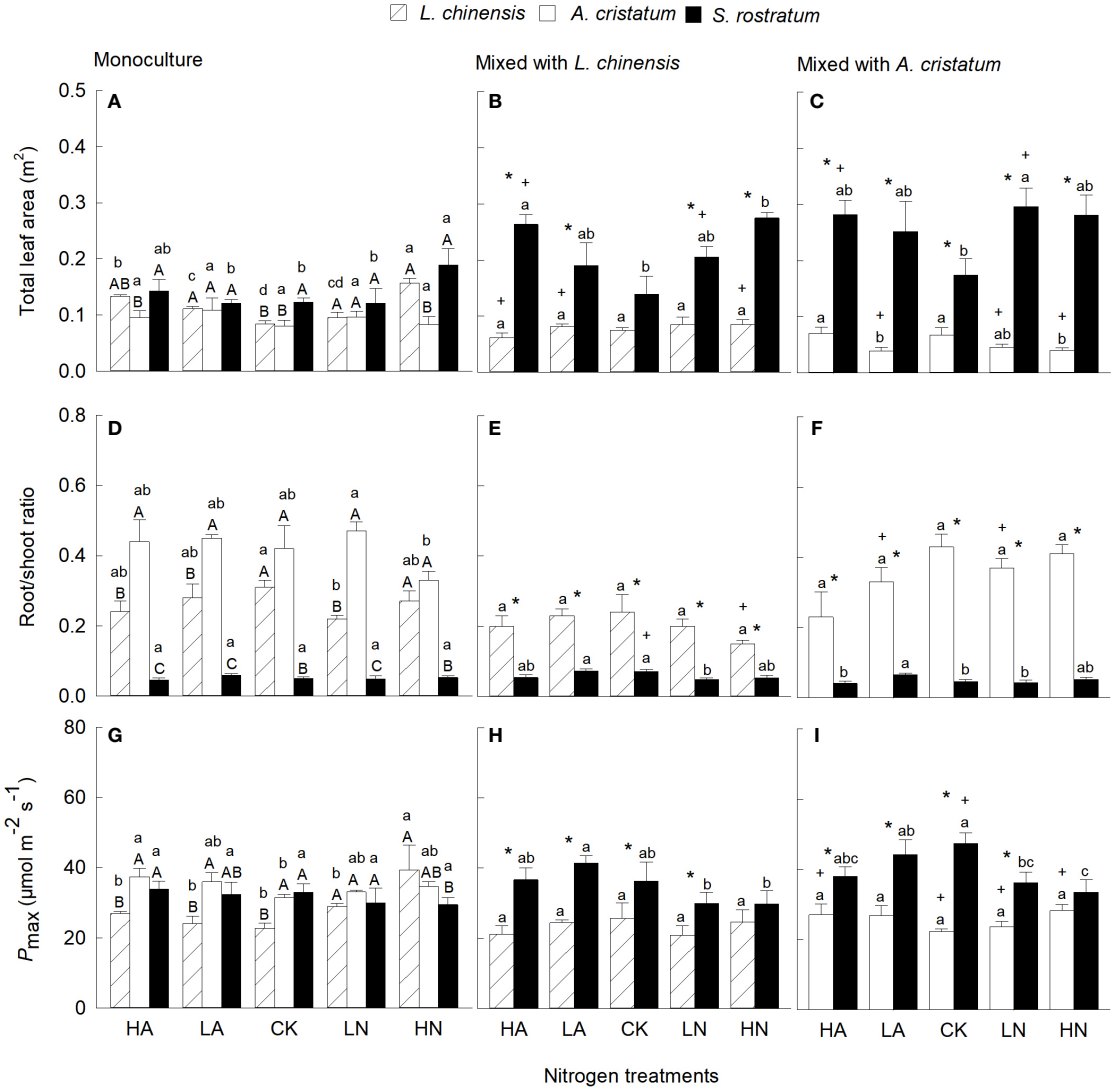
Total leaf area **(A–C)**, root/shoot ratio **(D–F)** and light-saturated net photosynthetic rate (G–I; Pmax) for Leymus chinensis, Agropyron cristatum and Solanum rostratum grown in mono- **(A, D, G)** and mixed **(B, C, E, F, H, I)** cultures under different nitrogen treatments. HA, high ammonium; LA, low ammonium; CK, control; LN, low nitrate; HN, high nitrate. Mean ± SE (n = 4). Different upper- (* in mixed culture; P < 0.05, independent samples t-test) and lowercase letters indicate significant differences between species under the same nitrogen treatment and those between nitrogen treatments for the same species under the same planting method, respectively (P < 0.05; one-way ANOVA). + indicates significant difference between the mixed and monocultures under the same nitrogen treatment (P < 0.05, independent samples t-test).

Total leaf area of the invader was significantly higher than that of the two native plants in mixed culture (except mixed with *L. chinensis* under CK), but the differences were not always significant in monoculture (**Figures 3A−C**). Consistently, our four-way ANOVA also showed that species and their interaction with planting method significantly affected total leaf area (**Table S1**). Compared with CK, N addition increased total leaf area for *S. rostratum* and *L. chinensis*, but not for *A. cristatum* in monoculture. In mixed culture, however, N addition increased total leaf area for the invader, but not for the two native plants, increasing the magnitude of the difference in total leaf area between the invasive and native plants. (**Figures 3B, C**). Compared with monoculture, mixed culture increased total leaf area for the invader, while reduced total leaf area for the native plants, increasing the differences between the invasive and native plants.

Root to shoot ratio was significantly lower in the invasive relative to native plants under all N treatments in both mixed and monocultures (**Figures 3D−F**). Compared with monoculture, mixed culture reduced root to shoot ratio for the two native plants (except *A. cristatum* under CK and high ammonium), but not for the invader. The effects of N levels on root to shoot ratio were not significant, which was consistent with the results of our four-way ANOVA (**Table S1**).

When grown in monoculture, *P*_max_ was significantly higher for the invader than for *L. chinensis* under CK and high ammonium, but similar for the invader and *A. cristatum* under all N treatments (**Figures 3G−I**). When grown in mixed culture, *P*_max_ was significantly higher for the invader than for the two natives under all N treatments except high nitrate. Compared with monoculture, mixed culture promoted *P*_max_ for the invader, but decreased *P*_max_ for *L. chinensis* (except under CK and low ammonium) and *A cristatum* under all N treatments. Our four-way ANOVA also showed that species and their interaction with planting method significantly affected *P*_max_ (**Table S1**).

### Effects of total leaf area, root to shoot ratio on growth

**Figure 4:**
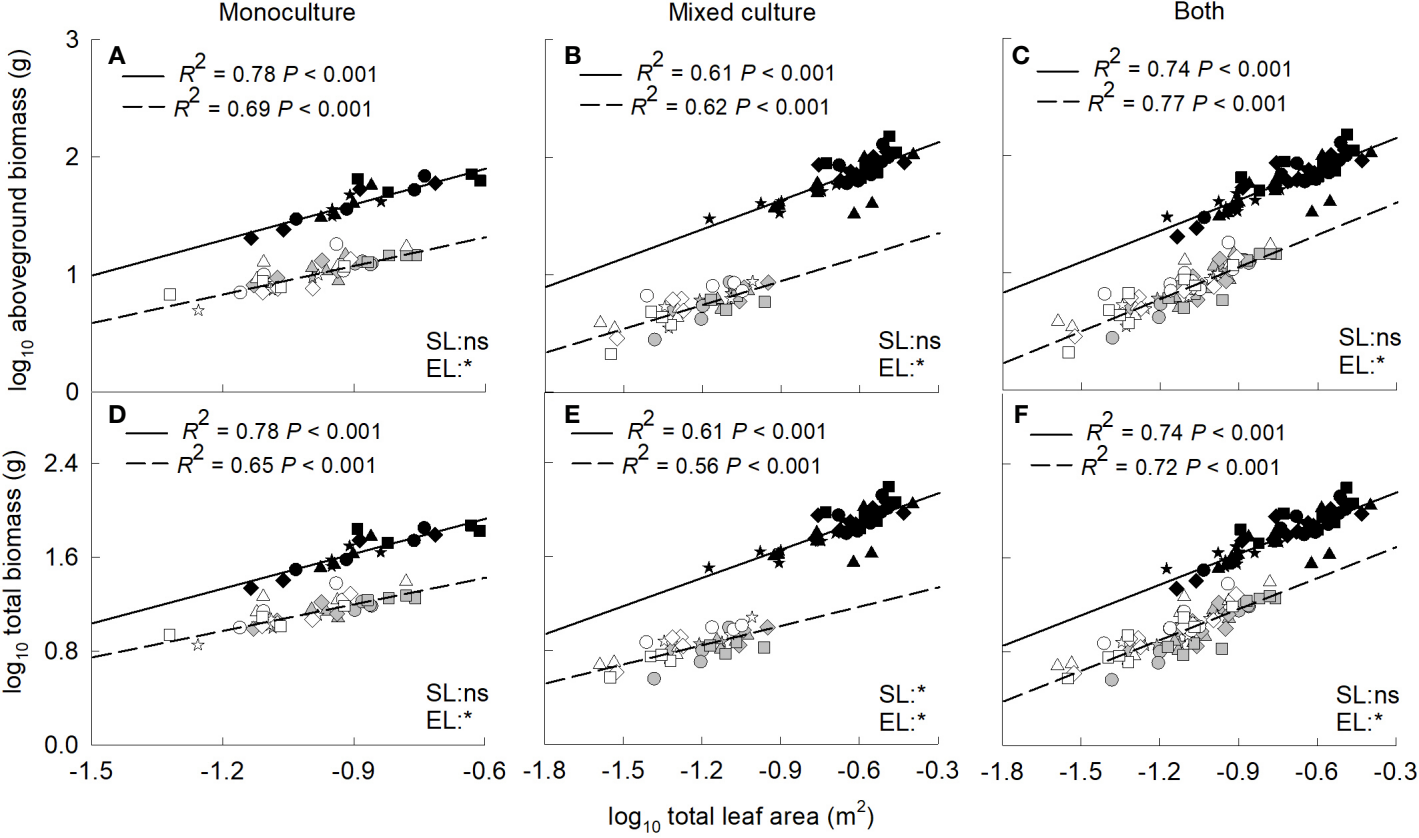
Standardized major axis regressions between aboveground biomass **(A–C)**, total biomass **(D–F)** and total leaf area for the invasive (solid line) and the two native (dashed line) species grown in mono- **(A, D)** and mixed **(B, E)** cultures and both **(C, F)** under different nitrogen treatments. SL, slope; EL, intercept. *, significant differences (*P* < 0.05); ns, non-significant differences. Gray symbols, *Leymus chinensis*; open symbols, *Agropyron cristatum*; closed symbols, *Solanum rostratum*. Stars, control; diamonds, low nitrate; squares, high nitrate; triangles, low ammonium; circles, high ammonium. The R^2^ was indicated on the figure, followed by the *P*-values.

**Figure 5:**
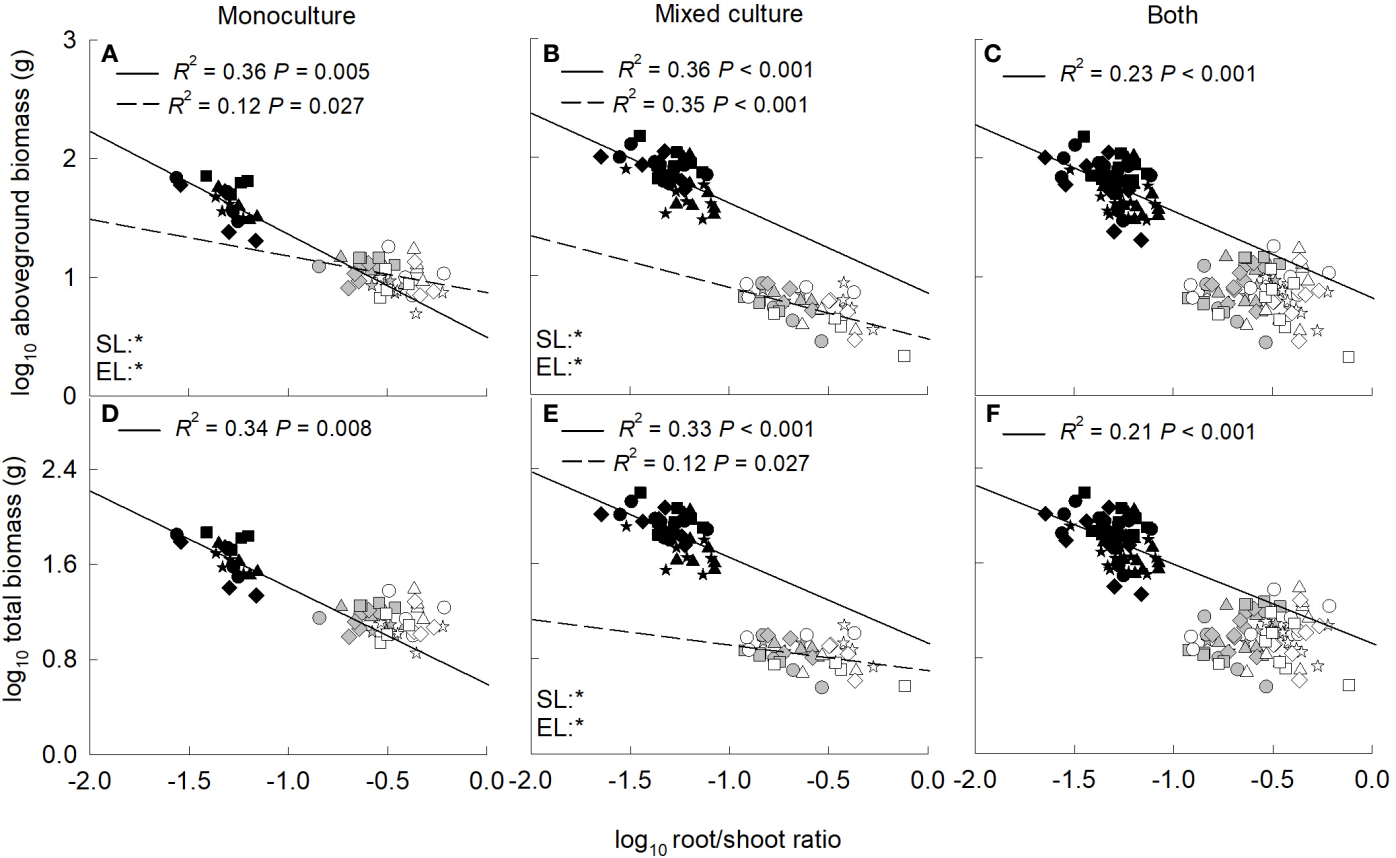
Standardized major axis regressions between aboveground biomass **(A–C)**, total biomass **(D−F)** and root/shoot ratio for the invasive (solid line) and the two native (dashed line) species grown in mono- **(A, D)** and mixed culture **(B, E)** and both **(C, F)** under different nitrogen treatments. SL, slope; EL, intercept. *, significant differences (*P *< 0.05); ns, non-significant differences. Gray symbols, *Leymus chinensis*; open symbols, *Agropyron cristatum*; closed symbols, *Solanum rostratum*. Stars, control; diamonds, low nitrate; squares, high nitrate; triangles, low ammonium; circles, high ammonium. The R^2^ was indicated on the figure, followed by the *P*-values.

Aboveground and total biomass increased significantly with the increase of total leaf area for the invasive and native plants when grown in monoculture, mixed culture or both (**Figure 4**). In addition, the biomass was significantly higher for the invasive relative to the native plants at the same value of total leaf area. In contrast, aboveground and total biomass decreased significantly with the increase of root to shoot ratio for the invader when grown in monoculture, mixed culture or both (**Figure 5**). For the two native plants, the aboveground biomass in monoculture, and the aboveground and total biomass in mixed culture were also negatively correlated with root to shoot ratio (**Figures 5A, B, E**).

## Discussion

Our study showed that the invasive plant *S. rostratum* had higher biomass and competitive ability than the native plants *L. chinensis* and *A. cristatum* under all N levels and in both mixed and monocultures, and N addition enhanced the advantages of the invader under most conditions. The invader’s advantages were associated with its high total leaf area and low root to shoot ratio. Extensive studies have shown that the increase of soil N availability often facilitates invasion success of exotic plants (Liao et al., 2013; Qin et al., 2013; Zhang et al., 2017; Musso et al., 2021). In addition, our study showed that a yearly increase in the nitrate to ammonium ratio of aerially deposited N may also promote the successful invasion of *S. rostratum*.

### Growth and competitive ability

Consistent with our hypothesis, the growth and competitive ability of the invader were superior to those of the native plants in both mixed and monocultures, and N addition was beneficial to the invader. The stronger competitive ability may help the invader to compete for resources, promoting its growth and invasion (Zheng et al., 2015; Liang et al., 2020). In contrast to the invader, N addition did not significantly influence the growth of *L. chinensis*, and even reduced the growth of *A. cristatum* in mixed culture, although promoting their growth in monoculture, decreasing their competitive abilities. These findings indicate that planting methods modify the effects of N addition on growth (Liang et al., 2020; Elias and Agrawal, 2021). In mixed culture, the native plants responded to N addition much more weakly than the invader, and also than itself in monoculture, indicating that more added N was used by the invader. In addition, N addition increased total leaf area of the invader, shading the native plants, which may be another reason for the smaller response of the native plants to N addition (Feng et al., 2007b; Zheng et al., 2009). In monoculture, the two native plants had a lower plastic response to N addition than the invader, which might be attributed to their long-term adaptation to arid/semi-arid barren grasslands (Wang et al., 2022).

N addition enhanced the growth and competitive advantages of the invader over the native plants under most conditions, which was consistent with the findings on other invasive plants (Liao et al., 2013; Qin et al., 2013; Jia et al., 2016; Zhang et al., 2017). For example, Zhang et al. (2017) found that N addition enhanced the competitive advantage of the exotic plant *Alternanthera philoxeroides* over the native plants *Oenanthe javanica* and *Iris pseudacorus*. However, these studies are mostly on the exotic plants in humid and fertile habitats, whereas *S. rostratum* mainly invades arid/semi-arid and barren habitats (Feng, 2020). Our study suggests that the invasion of exotic plants in barren habitats may also increase in the context of global changes such as N deposition. Leishman and Thomson (2005) also found that N addition promoted invasion of exotic plants in low-fertile soils.

Consistent with our hypothesis, growth and competitive ability were significantly higher for *S. rostratum* under low nitrate relative to low ammonium when mixed with *L. chinensis*. This result indicate that the invader prefers nitrate relative to ammonium, in line with the result from our research using ^15^N labeling (data not shown). No N form preference, however, was found for *S. rostratum* in monoculture, mixed with *A. cristatum*, and even mixed with *L. chinensis* under high N. In addition, the growth and competitive abilities of the two native plants were similar under nitrate and ammonium N, showing no N form preference. Similar results were also found for grasses after 6 years of N addition (Song et al., 2012). Song et al. (2012) found that aboveground biomass of grasses were not significant difference between ammonium N and nitrate N after 6 years of N addition. Our results showed that species, soil N levels, planting methods, and competition all affected N form preferences. Plants also have plasticity in N form acquisition (Hu et al., 2019; Zhang et al., 2018; Qian et al., 2021; Sun et al., 2021). For example, the dominant species *Kobresia myosuroides* in alpine meadow prefers nitrate N in monoculture, but ammonium when mixed with the non-dominant species *Mertensia lanceolate* (Ashton et al., 2010). Hu et al. (2019) found that the N forms preferred by *Chromolaena odorata* and *Ageratina adenophora* shifted from nitrate to ammonium with the increase of their invasion degree. N form preference of *S. rostratum* disappeared under high N level when mixed with *L. chinensis*, which may be associated with the compensatory effects of high ammonium for its relatively low uptake rate.

### Traits contributing to the superiority of the invader

Consistent with our hypothesis, total leaf area was significantly higher for the invasive relative to the native plants, and the interspecific difference was more pronounced in mixed relative to monoculture. These results demonstrated that the invader had a stronger ability to capture aboveground resources, which may be an essential factor for its successful invasion. In addition, the higher total leaf area was also beneficial to the invader by shading native plants (Feng et al., 2007b). Zheng et al. (2009) also found that total leaf area of the invasive plant *A. adenophora* was significantly higher than that of its co-occurring native plants. Our study further found that both aboveground biomass and total biomass were positively correlated with total leaf area. In addition, the invader had significantly higher biomass than the native plants at the same value of total leaf area, which may be associated with the higher *P*_max_ of the invader (especially in mixed culture). The higher *P*_max_ of the invasive relative to native plants enabled it to accumulate more biomass under the same leaf area. The positive correlation between biomass and *P*_max_ has also been reported in literatures (Zheng et al., 2009; Honda et al., 2021). High *P*_max_ may be a common feature of invasive plants (Liu et al., 2022).

Compared with *L. chinensis*, and *A. cristatum*, the invader had lower root to shoot ratio, which may be another reason for its higher biomass. Low root to shoot ratio can reduce root respiratory carbon consumption (Zheng et al., 2009), and leave more biomass for leaves and support organs, increasing aboveground light energy capture and promoting exotic plant invasions (Zheng et al., 2009; Liao et al., 2013). In addition, low root to shoot ratio can also decrease total root biomass, the amount of organic matter released from roots to soils (He et al., 2017), and thus root carbon loss. Negative correlation between biomass and root to shoot ratio was indeed found in our study. It has been showed that low root to shoot ratio facilitated the invasion by *C. odorata* in fertile habitats, but adversely affected its invasion in barren habitats (Liao et al., 2013; Qin et al., 2013). In the present study, soil nutrient contents were high (see “Study sites and species”), and thus the lower root to shoot ratio contributed to invasion of the invader. On the other hand, high nutrient of the habitat usually can improve plant water resource uptake and use, which may be benefit for *S. rostratum* adapt arid/semi-arid and barren habitats.

It has been found that soil nutrient availability affects the effect of N addition (Xu et al., 2014; Ye et al., 2022). Xu et al. (2014) found that the response of plant yield to N addition was higher in barren soil than in fertile soil. Thus, the results of our study conducted in the fertile soil may not truly reflect the response of *S. rostratum* to atmospheric N deposition in other habitats with relatively low soil nutrients. Further studies on the effects of N addition and related mechanisms are needed for *S. rostratum* in other habitats with different soil nutrients.

## Conclusion

Our study showed that growth and competitive ability were significantly higher for the invasive plant *S. rostratum* than for *L. chinensis*, and *A. cristatum* (natives) in most cases, and N addition increased the advantages of the invader, contributing to its invasion success. Nitrate relative to ammonium was more beneficial to invasion of *S. rostratum* at low N levels. The growth and competitive advantages of *S. rostratum* were associated with its higher total leaf area and lower root to shoot ratio. Our results indicated that N deposition, especially nitrate N deposition, may also promote invasions of barren-tolerant exotic plants such as *S. rostratum* in arid/semi-arid and barren habitats. The effects of N forms and interspecific competition, which can alter not only plant N form preferences but also interspecific differences in functional traits, also need to be taken into consideration when studying the effects of N deposition on the invasiveness of exotic plants.

## Data availability statement

The original contributions presented in the study are included in the article/**Supplementary Material**. Further inquiries can be directed to the corresponding authors.

## Author contributions

J-KS: performing experiments, data analysis, and writing of manuscript. K-QT, E-XT, J-MC and X-RL: performing experiments. M-CL and Z-XL: conceptualization, and performing some of the experiments. Y-LF: conceptualization, supervision, data analysis, and writing of manuscript. All authors contributed to the article and approved the submitted version.
