# Supplementary material for: Advantages of growth and competitive ability of the invasive plant Solanum rostratum over two co-occurring natives and the effects of nitrogen levels and forms
Source: Front Plant Sci. 2023 Apr 18;14:1169317. doi: 10.3389/fpls.2023.1169317 (PMC10151799; doi:10.3389/fpls.2023.1169317)
Supplement: Supplementary file 1 [file DataSheet_1.pdf]

Supplementary material

Table S1 Effects of species (*n* = 3; S), nitrogen forms (*n* = 2; NF), nitrogen levels (*n* = 2; NL), planting methods (*n* = 2; PM) and their interactions on aboveground biomass (AB), total biomass (TB), root to shoot ratio (R/S), total leaf area (TLA), and maximum net photosynthetic rate (P_{max}).

Source of		AB		TB		R/S		TLA		P_{max}	
variation	*df*	*F*	*P*	*F*	*P*	*F*	*P*	*F*	*P*	*F*	*P*
S	2	**168.2**	**< 0.001**	**162.2**	**< 0.001**	**202.2**	**< 0.001**	**87.6**	**< 0.001**	**14.7**	**< 0.001**
NF	1	1.2	0.278	1.0	0.319	0.0	0.998	0.4	0.513	0.9	0.344
NL	1	**4.1**	**0.046**	3.9	0.051	3.6	0.062	**5.0**	**0.028**	1.3	0.254
PM	1	**5.5**	**0.022**	3.8	0.056	**12.3**	**0.001**	0.6	0.455	**10.3**	**0.002**
S × NF	2	2.2	0.112	2.4	0.095	1.6	0.212	1.2	0.292	**6.5**	**0.003**
S × NL	2	**3.9**	**0.025**	**4.0**	**0.021**	1.0	0.376	2.2	0.112	1.8	0.176
S × PM	2	**20.0**	**< 0.001**	**23.1**	**< 0.001**	**4.1**	**0.021**	**41.8**	**< 0.001**	**15.2**	**< 0.001**
NF × NL	1	0.0	0.962	0.0	0.968	0.6	0.450	0.2	0.669	2.3	0.129
NF × PM	1	0.3	0.582	0.4	0.539	1.9	0.172	0.0	0.929	2.6	0.111
NL × PM	1	0.0	0.881	0.0	0.894	0.0	0.989	0.4	0.548	1.8	0.180
S × NF × NL	2	0.0	0.979	0.0	0.956	0.1	0.944	0.6	0.541	0.7	0.499
S × NF × PM	2	0.2	0.853	0.1	0.875	**4.5**	**0.014**	0.1	0.928	1.6	0.206
S × NL × PM	2	0.1	0.864	0.2	0.824	1.0	0.389	1.6	0.211	0.8	0.455
NF × NL × PM	1	1.4	0.239	1.5	0.222	1.0	0.336	1.5	0.218	0.5	0.489
S × NF × NL × PM	2	0.8	0.474	0.7	0.504	**3.9**	**0.024**	0.2	0.811	0.2	0.839

Whereas, the significant effects are shown in bold letters.

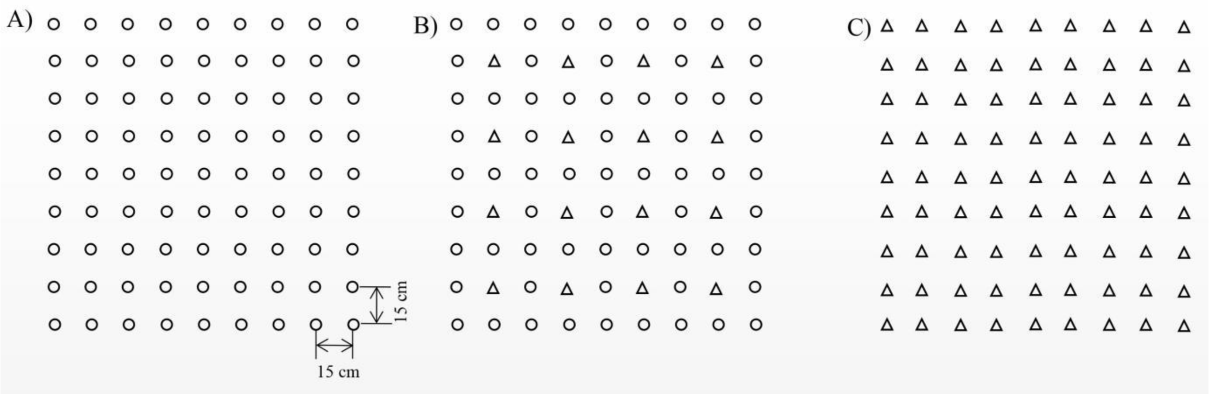

Figure S1 Schematic illustration of plant cultivation mode. A, monoculture of *Leymus chinensis* or *Agropyron cristatum*; B, mixed culture of *L. chinensis* or *A. cristatum* with *Solanum rostratum*; C, monoculture of *S. rostratum*. Plants spaced at a distance of 15 cm.
